# Supplementary material for: The rising moon promotes mate finding in moths
Source: Commun Biol. 2022 Apr 28;5:393. doi: 10.1038/s42003-022-03331-x (PMC9051113; doi:10.1038/s42003-022-03331-x)
Supplement: Supplementary file 2 — Supplementary Information [file 42003_2022_3331_MOESM2_ESM.pdf]

Supplementary Information for: The rising moon promotes mate finding in moths

Supplementary Results and Discussion

The animals tested in the experiment differed in size. We included the robust factor “forewing length” for moth sizes in the model and were unable to detect any effect of body size on flight success (arrival at a trap; logistic regression: z=1.21, *p*=0.228, *n*=58) or flight duration (GAM Cox PH, *z*=-0.45, *p*=0.652, *n*=34). In addition to individuals from the breeding we tested wild caught animals to assure that the results from breeding animals are representative. As expected, breeding animals and wild-caught animals did not differ significantly in arrival (logistic regression: z=-1.46, *p*=0.146, *n*=58) and survival analysis (GAM Cox PH, z=0.78, *p*=0.436, *n*=34).

A

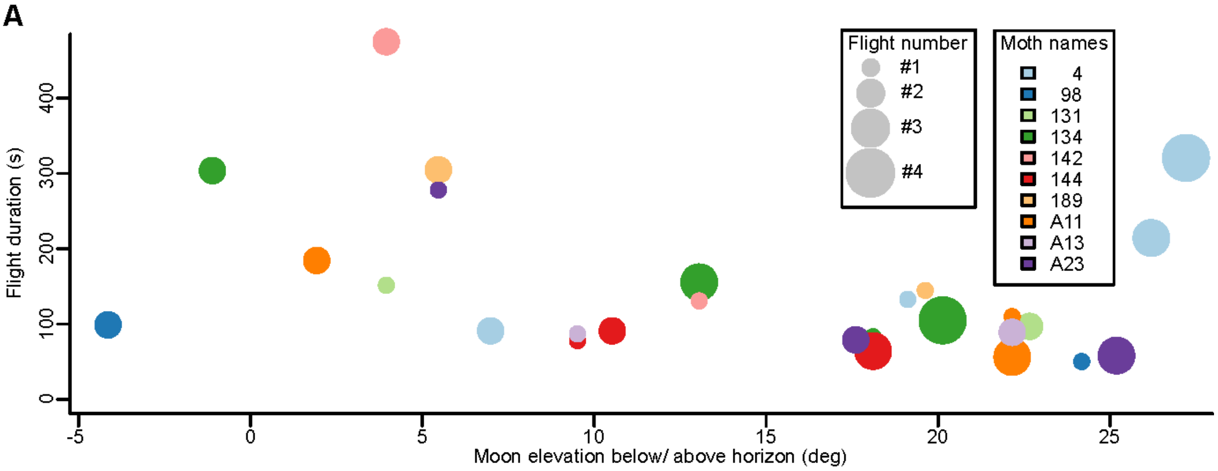

Fig. S1 Flight duration of individuals and the number of flights performed. Each colour marks a single individual with consecutive flights – where applicable – represented by the size of the dots, increasing from the first to the fourth flight. Please note that, different to Fig. 1, only flights of individuals with more than one flight are displayed in this figure.

To exclude the possibility that males with multiple releases reached a trap progressively faster due to their increasing experience, we analysed the data of males with more than one flight on an individual basis (Fig. S1). If experience matters, one would expect longer flight durations of first flights compared to subsequent flights. However, the opposite was true for some animals (Fig. S1, moths 98 and 189) and flight durations also fluctuated from faster to slower and back to faster (Fig. S1, moths 134 and 144) or vice versa (Fig. S1, moth A11). Thus, the significant decrease in flight duration (Fig. 1) cannot be explained by the sequence of flights performed by an individual. Note, that this was already considered in the model via a potential random effect of each individual. However, Fig. S1 underpins this result in a descriptive and intuitive way. Interestingly, the two outliers in flight duration for a moon elevation higher than 25° go back to one individual (Fig. S1, moth 4). It is therefore likely that this individual took longer to reach a trap than the other males due to unknown reasons related only to this animal.
