# Supplementary material for: The rising moon promotes mate finding in moths
Source: Commun Biol. 2022 Apr 28;5:393. doi: 10.1038/s42003-022-03331-x (PMC9051113; doi:10.1038/s42003-022-03331-x)
Supplement: Supplementary file 3 — Reporting Summary [file 42003_2022_3331_MOESM3_ESM.pdf]

nature portfolio

Corresponding author(s): Jacqueline Degen

Last updated by author(s): Feb 25, 2022

Reporting Summary

Nature Portfolio wishes to improve the reproducibility of the work that we publish. This form provides structure for consistency and transparency in reporting. For further information on Nature Portfolio policies, see our Editorial Policies and the Editorial Policy Checklist.

Statistics

For all statistical analyses, confirm that the following items are present in the figure legend, table legend, main text, or Methods section.

n/a	Confirmed	
☐	☒	The exact sample size (*n*) for each experimental group/condition, given as a discrete number and unit of measurement
☐	☒	A statement on whether measurements were taken from distinct samples or whether the same sample was measured repeatedly
☐	☒	The statistical test(s) used AND whether they are one- or two-sided<br>*Only common tests should be described solely by name; describe more complex techniques in the Methods section.*
☐	☒	A description of all covariates tested
☐	☒	A description of any assumptions or corrections, such as tests of normality and adjustment for multiple comparisons
☐	☒	A full description of the statistical parameters including central tendency (e.g. means) or other basic estimates (e.g. regression coefficient) AND variation (e.g. standard deviation) or associated estimates of uncertainty (e.g. confidence intervals)
☐	☒	For null hypothesis testing, the test statistic (e.g. *F*, *t*, *r*) with confidence intervals, effect sizes, degrees of freedom and *P* value noted<br>*Give P values as exact values whenever suitable.*
☒	☐	For Bayesian analysis, information on the choice of priors and Markov chain Monte Carlo settings
☒	☐	For hierarchical and complex designs, identification of the appropriate level for tests and full reporting of outcomes
☒	☐	Estimates of effect sizes (e.g. Cohen's *d*, Pearson's *r*), indicating how they were calculated

Our web collection on statistics for biologists contains articles on many of the points above.

Software and code

Policy information about availability of computer code

Data collection	No computer code was needed.
Data analysis	R statistical programming environment version 4.0.3<br>Function glmer from R package lme4, version 1.1-26<br>Function gam from R package mgcv, version 1.8-33, and link function cox.ph

For manuscripts utilizing custom algorithms or software that are central to the research but not yet described in published literature, software must be made available to editors and reviewers. We strongly encourage code deposition in a community repository (e.g. GitHub). See the Nature Portfolio guidelines for submitting code & software for further information.

Data

Policy information about availability of data

All manuscripts must include a data availability statement. This statement should provide the following information, where applicable:

- Accession codes, unique identifiers, or web links for publicly available datasets
- A description of any restrictions on data availability
- For clinical datasets or third party data, please ensure that the statement adheres to our policy

The dataset used in this study as well as the raw and processed all-sky pictures are available on DRYAD at DOI https://doi.org/10.5061/dryad.wdbrv15qn.

Field-specific reporting

Please select the one below that is the best fit for your research. If you are not sure, read the appropriate sections before making your selection.

☐ Life sciences ☐ Behavioural & social sciences ☒ Ecological, evolutionary & environmental sciences

For a reference copy of the document with all sections, see nature.com/documents/nr-reporting-summary-flat.pdf

Ecological, evolutionary & environmental sciences study design

All studies must disclose on these points even when the disclosure is negative.

Study description	We monitored a total of 58 flights of free flying moths to investigate whether they reach females located north or south of the release site within the experimental time of eight minutes. If they reached the females within this time, the exact flight duration was measured. For all males that reached the females, flights were linked to all-sky pictures taken during the flight for quantification of the light environment. Besides the flight duration, the choice (north or south) was analysed with respect to the light environment including both, natural and artificial light sources.
Research sample	Privet hawk moth males (Sphinx ligustri L., Lepidoptera, Sphingidae) originating from a breeding as well as freshly caught ones were tested.
Sampling strategy	Since the present study was a field research study, sample size was determined by animal availability and weather conditions.
Data collection	We recorded the data with a small team of three people (M.S., A.Ja., J.D.). Flight data were recorded with very basic equipment (a stopwatch) and sky pictures were taken using the established method of all-sky photometry.
Timing and spatial scale	The study was conducted from 19 July 2019 to 31 July 2019 . We sampled as many individuals as possible (restricted by the total number available) in as many night hours as possible (restricted by weather contidions).
Data exclusions	No data were excluded from the analysis.
Reproducibility	The experiment has not been repeated but was conducted for slowly changing light conditions at the experimental field close to Marburg, Germany.
Randomization	Each experimental animal available was tested once each experimental day.
Blinding	Blinding was not relevant to the study because we did not interfere with the behavior of animals during the experiments. The animals were prepared during the day and therefore well in advance of release. They were stored beneath tins, sitting on small wooden plates. The plate as well as the animal below the tin were placed on the release table and the tin was removed to set the animal free. We did not interfere anymore with behavior, the animals started on their own to vibrate and fly. Once they arrived at one of the cages with females, the experiment was complete for that day and the flight duration was measured. However, not all animals were treated like this. We described other handling procedures in the manuscript, but since we found no significant differendes between groups, the handling procedure did not significantly affect behavior.

Did the study involve field work? ☒ Yes ☐ No

Field work, collection and transport

Field conditions	Experiments were only performed during warm summer nights without rain or strong wind.
Location	The study was conducted on a meadow located east of Großseelheim and south of the river Ohm in the German State Hesse (50° 49'17.5"N 8°52'15.4"E). This area had a relatively low impact of ALAN on the night sky brightness at zenith (0.34 mcd/m²).
Access & import/export	We obtained permission for capture and release from the Regional Council of Giessen, Germany, 26.04.2019.
Disturbance	We kept the disturbance as low as possible by adjusting the time of experiments to the breeding of protected birds in that area.

Reporting for specific materials, systems and methods

We require information from authors about some types of materials, experimental systems and methods used in many studies. Here, indicate whether each material, system or method listed is relevant to your study. If you are not sure if a list item applies to your research, read the appropriate section before selecting a response.

Materials & experimental systems

n/a	Involved in the study	
☒	☐	Antibodies
☒	☐	Eukaryotic cell lines
☒	☐	Palaeontology and archaeology
☐	☒	Animals and other organisms
☒	☐	Human research participants
☒	☐	Clinical data
☒	☐	Dual use research of concern

Methods

n/a	Involved in the study	
☒	☐	ChIP-seq
☒	☐	Flow cytometry
☒	☐	MRI-based neuroimaging

Animals and other organisms

Policy information about studies involving animals; ARRIVE guidelines recommended for reporting animal research

Laboratory animals	We reared moths (Sphinx ligustri L., Lepidoptera, Sphingidae) that derived from a mated female caught in 2018 at the same experimental field in Großseelheim.
Wild animals	All wild animals captured in 2019 were attracted by the caged females. They were treated the same way as animals from the breeding afterwards. All animals were released when the experimental period was over.
Field-collected samples	Pupaes were stored in the fridge at 5°C. Afterwards they were kept at room conditions.
Ethics oversight	We obtained permission for capture and release from the Regional Council of Giessen, Germany.

Note that full information on the approval of the study protocol must also be provided in the manuscript.
